# Case Report: Two cases of staged combined treatment for complex skull-exposing wounds: synergistic effects of mechanical tension and moist wound healing

**DOI:** 10.3389/fmed.2025.1670005

**Published:** 2025-09-09

**Authors:** Jianqiong Lin, Yanfei Ma, Xiaohong Zeng, Yan Zhang, Hongyuan Liu

**Affiliations:** Mianyang Central Hospital, School of Medicine, University of Electronic Science and Technology of China, Mianyang, Sichuan, China

**Keywords:** skull exposure, staged therapy, sustained skin-stretching device, moist wound healing, complex cranial wounds

## Abstract

Complex skull-exposing wounds complicated by repeated surgical failures, implant-associated infections, and a history of targeted drug therapy present substantial challenges for reconstruction. This study retrospectively analyzed two such cases to evaluate the clinical outcomes of a staged treatment strategy integrating a sustained skin-stretching device (SSD) with moist wound therapy. Case 1, with four previous failed surgeries, achieved complete closure within 40 days, with no recurrence during 6 months of follow-up. Case 2, after three debridement procedures, achieved closure in 45 days with stable scar formation and no dehiscence at 6 months. The treatment protocol incorporated SSD with silver ion dressings, recombinant human basic fibroblast growth factor (rh-bFGF), mussel adhesive protein-based dressings, and Moist Exposed Burn Ointment (MEBO), supplemented by surgical debridement as required. Controlled mechanical tension and an optimized moist microenvironment promoted progressive wound edge advancement, effective infection control, and tissue regeneration. The findings indicate that staged mechanical traction combined with moist wound dressings may represent a minimally invasive and effective approach for managing complex cranial wounds.

## 1 Introduction

Skull exposure refers to the direct uncovering of cranial bone resulting from various causes such as trauma or pathological conditions, characterized by the loss of overlying scalp tissue and visible irregular bony contours (1, 2). It most frequently involves the vertex but can also occur in the frontal or temporal regions (3). When accompanied by chronic wounds, skull exposure is often associated with impaired healing due to factors such as repeated surgical failure, implant-related infection, or systemic conditions including malnutrition and targeted anticancer therapies. In cases where bone is exposed and conventional treatments fail to achieve closure, wound management becomes particularly challenging. Traditional approaches such as flap transfer or skin grafting can offer coverage, but their applicability is often limited in patients with chronic wounds, compromised local tissue conditions, or poor surgical tolerance. In recent years, wound care strategies have shifted from passive coverage to active regeneration. The sustained SSD, as a novel technique, exerts continuous and adjustable mechanical tension to promote gradual skin edge advancement and tissue expansion, facilitating spontaneous wound closure (4, 5). At the same time, moist wound dressings help maintain an optimal wound environment by preserving hydration, supporting cell proliferation, and accelerating healing. They are particularly beneficial in modulating the local microenvironment, minimizing pain, and preventing infection (6, 7).

This study presents two cases of skull-exposed chronic non-healing wounds. Both patients had undergone multiple surgical interventions complicated by infection and diminished cellular activity, leading to limited efficacy of mechanical traction alone. By incorporating moist wound dressings—including silver ion dressings, recombinant human basic fibroblast growth factor (rh-bFGF), mussel adhesive protein-based dressings, and Moist Exposed Burn Ointment (MEBO)—the wound microenvironment was progressively modulated in a staged manner, ultimately achieving complete wound closure. These cases highlight a potentially effective treatment strategy and may offer clinical insight into the management of similarly challenging skull-exposing wounds.

## 2 Case presentation

### 2.1 Case 1

Patient Information: A 36-year-old male was admitted for “recurrent non-healing scalp wound for over 1 year.” The patient had a history of left renal clear cell carcinoma and was receiving oral targeted therapy. He had previously undergone four surgeries. The first surgery involved intracranial aneurysm clipping with decompressive craniectomy, after which the scalp healed well. Three months later, an autologous bone flap cranioplasty was performed, and the incision healed uneventfully.

**Figure 1:**
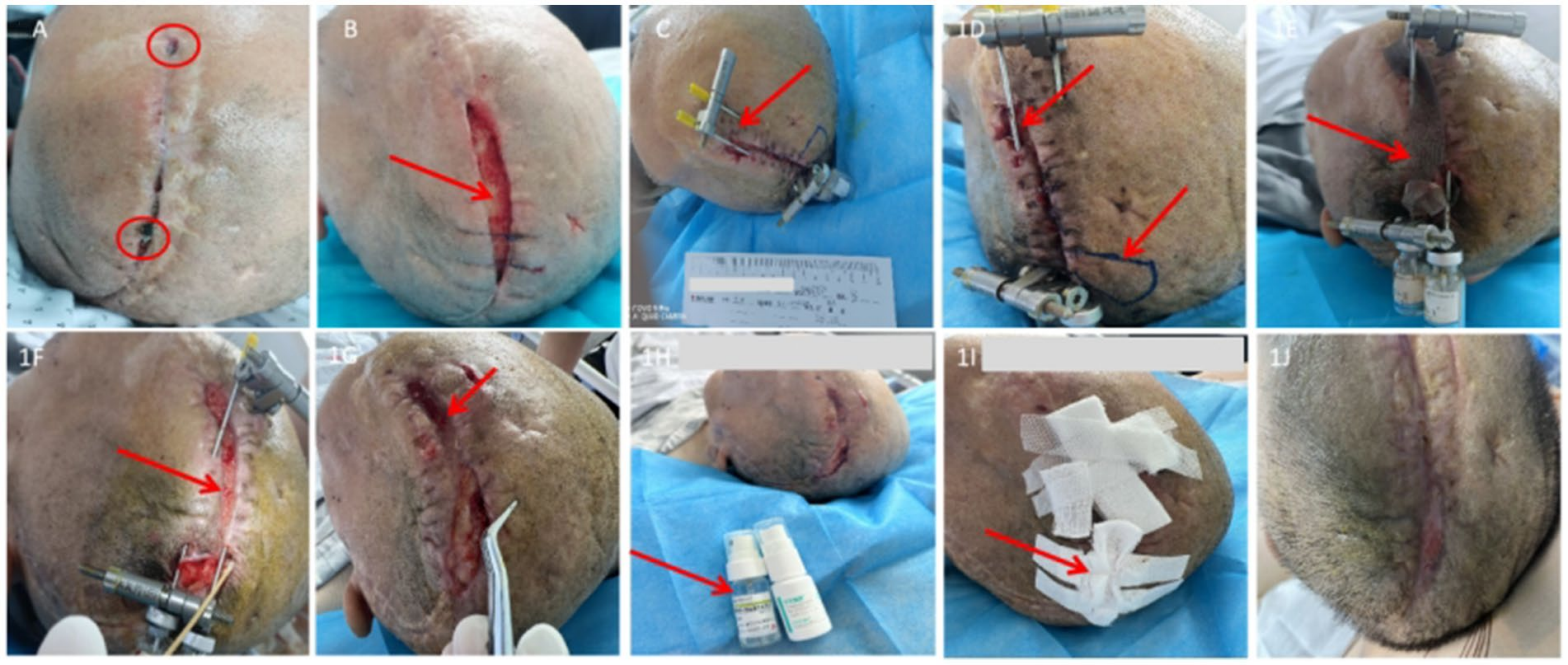
Case 1 wound progression. **(A)** Initial wound with multiple dehiscences (red circle indicates exposed titanium mesh). **(B)** After debridement, wound dehiscence persists (red arrow indicates exposed autologous cranial bone and poorly growing granulation tissue). **(C)** Post-placement of the distraction device, wound dehiscence recurs (red arrow indicates displacement of the distraction device). **(D)** Wound dehiscence with edge retraction (red arrow indicates exposed cranial bone and subcutaneous tunneling). **(E)** Gradual adjustment of the distraction device with moist dressing application (red arrow indicates silver ion moist dressing). **(F)** Discontinuation of silver ion dressing (red arrow indicates well-formed granulation tissue and reduced wound tension). **(G)** Removal of the distraction device (red arrow indicates fragile and irregular wound edges). **(H)** Application of moist dressings (recombinant human basic fibroblast growth factor and mussel adhesive wound repair dressing). **(I)** Coverage with MEBO ulcer dressing to maintain tension reduction (red arrow indicates self-adhesive elastic bandage approximating the wound edges). **(J)** Final wound closure.

One year after the autologous bone flap cranioplasty, most of the bone flap had resorbed. The patient underwent a “titanium mesh cranioplasty” through the original incision. Forty days postoperatively, crusting and dehiscence developed along a 25-cm-long surgical scar, with multiple wound openings and titanium mesh exposure. The largest wound measured 5 cm × 1 cm (Figure 1A). Despite active dressing changes and suturing, the wound failed to heal. Surgical debridement was performed through the original incision, removing the titanium mesh, necrotic bone, and foreign material.

Postoperatively, the incision showed poor healing with yellow exudate, mild local erythema and swelling, and small areas of yellow-white necrotic tissue. Debridement and silver ion dressings were applied. After 15 days of dressing changes, granulation tissue appeared red and healthy but grew slowly. The wound remained unhealed, with a 15 cm × 2 cm dehiscence and exposure of autologous cranial bone (Figure 1B).

The patient then underwent scalp debridement with primary suturing and placement of a sustained skin distraction (SSD) device. Three days postoperatively, distraction tension was gradually adjusted according to the condition of the scalp. Fifteen days after surgery, a 9.5 cm × 2 cm wound remained with exposed cranial bone. Subcutaneous tunneling of approximately 3 cm was observed, containing yellow-white necrotic tissue and foreign material (suspected autolyzed bone), along with device displacement and wound edge retraction (Figures 1C,D). Adjustment of the distraction device was halted, the final tension maintained, foreign material removed, and silver ion dressings applied (Figure 1E). Dressings were changed every 48 h. After achieving infection control, removing necrotic tissue, and observing a healthy wound bed, silver ion dressings were discontinued (Figure 1F), and the SSD device was removed. The surrounding skin remained fragile and irregular (Figure 1G), preventing further suturing.

Moist wound therapy was then applied: rh-bFGF was evenly sprayed onto the wound base and allowed to absorb for 5–10 min, followed by application of mussel adhesive wound repair dressing (Figure 1H), then covered with MEBO ulcer dressing. A self-adhesive elastic bandage was used to approximate the wound edges and maintain tension reduction (Figure 1I). After 40 days of combined mechanical distraction and moist wound therapy, the wound achieved complete closure (Figure 1J). At six-month follow-up, no recurrence was observed.

### 2.2 Case 2

**Figure 2:**
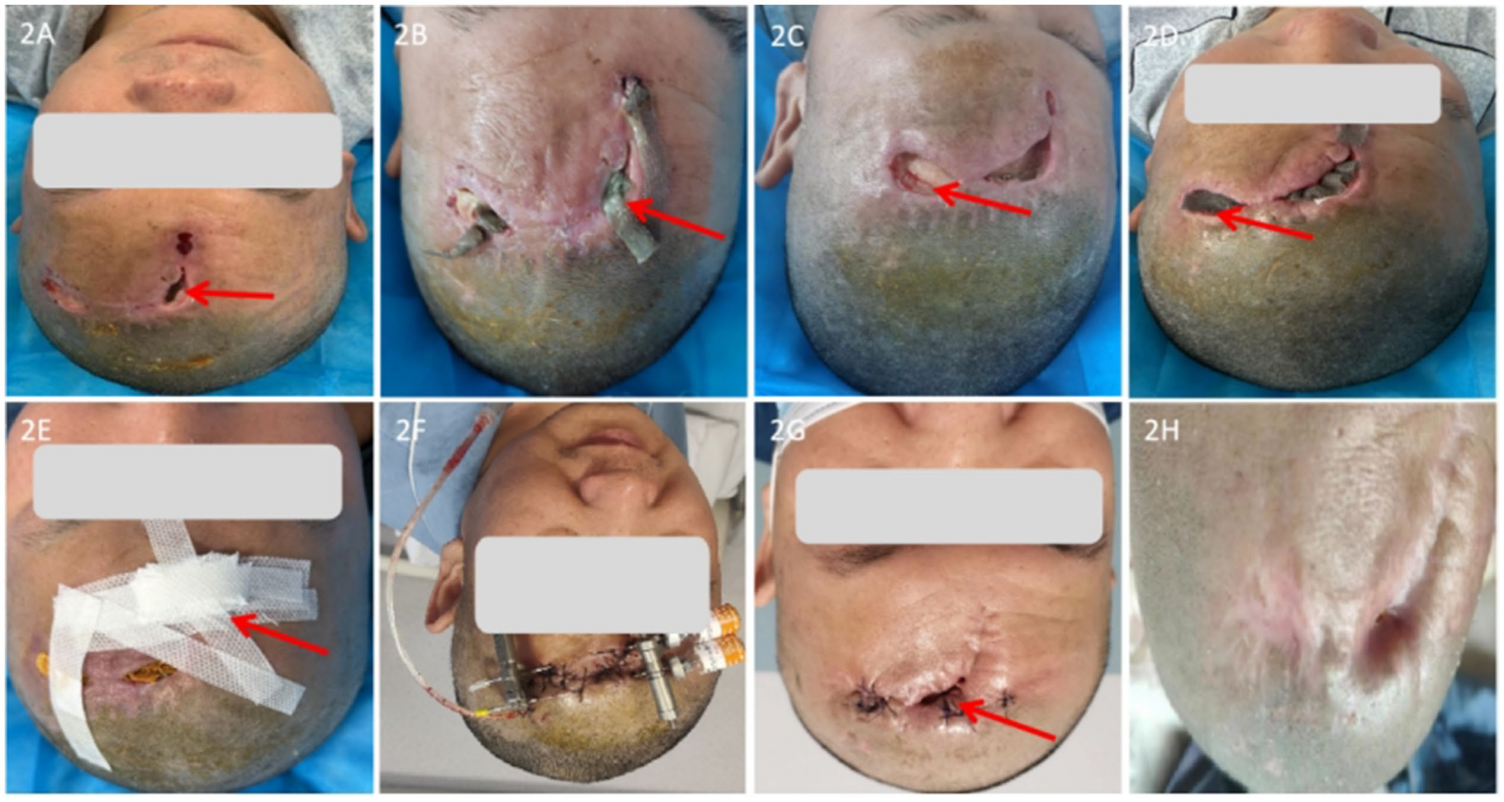
Case 2 wound progression. **(A)** Initial wound (red arrow indicates exposed cranial bone). **(B)** After first debridement (red arrow indicates purulent exudate). **(C)** After second surgery (red arrow indicates pale granulation tissue with poor vascularization). **(D)** Fragile wound edges with moist dressing changes (red arrow indicates silver ion dressing). **(E)** Approximation of wound edges. **(F)** Post-placement of the distraction device. **(G)** Rolled wound edges and subcutaneous tunneling. **(H)** Final wound closure.

Patient Information: A 50-year-old male with a history of lung cancer and brain metastasis, who had received postoperative radiotherapy and targeted therapy, was admitted for “left frontal craniotomy, internal decompression, and cerebrospinal fluid leak repair, followed by 1 month of frontal scalp infection with exposed cranial bone” (Figure 2A).

Upon admission, the patient underwent wound debridement and removal of implanted material. Ten days postoperatively, the wound remained unhealed with purulent exudate (Figure 2B). Three wound openings were observed, the largest measuring 2.5 cm × 1 cm × 0.8 cm, with visible bone exposure. Wound cultures grew methicillin-resistant *Staphylococcus aureus* (MRSA). Topical dressings containing gentamicin, metronidazole, and dexamethasone were applied, and targeted therapy was temporarily discontinued. After 15 days, the wound showed no signs of healing.

The patient underwent repeat debridement and external drainage. Ten days postoperatively, purulent exudate persisted. Silver ion dressings were applied, resulting in decreased exudate, but granulation tissue formation was slow, appeared pale, and was poorly vascularized. The wound openings increased in size and depth, with the largest measuring 3 cm × 1.8 cm × 1.0 cm. The wound edges were fragile. Dressing changes were used to approximate the wound edges (Figures 2C–E).

Subsequently, the patient underwent frontal wound debridement, skin distraction, and flap repair with drainage (Figure 2F). Three days postoperatively, the tension of the SSD device was gradually adjusted according to scalp condition. Fifteen days after surgery, the SSD device was removed, and a 2 cm × 0.8 cm wound remained unhealed, with rolled wound edges and subcutaneous tunneling up to 1 cm (Figure 2G). Rolled wound edges were trimmed, the subcutaneous tunnel was packed with silver ion dressing, rh-bFGF was evenly sprayed onto the wound base, followed by application of mussel adhesive wound repair dressing. MEBO ulcer dressing was applied, and a self-adhesive elastic bandage was used to approximate wound edges and maintain tension reduction.

After 45 days of combined mechanical distraction and moist wound therapy, the wound achieved complete closure (Figure 2H). At six-month follow-up, no recurrence was observed.

Note: Distraction device (SSD): Henan Keke Biotechnology Co., Ltd., China; Silver ion dressing: Bohmann Wet Silver, Germany; rh-bFGF: Gaifu, Nanhai Longtime Pharmaceutical Co., Ltd., China; Wound repair dressing: Youbeiruikang, Jiangyin Beirisen Biochemical Technology Co., Ltd., China (Table 1).

**Table 1:** Comparison of wound size, tunneling depth, dressing application, and healing time in the two cases.

Patient ID	Wound size		Tunneling depth (cm)	Distraction rate (mm/day) and duration	Dressing type and duration	Distraction–moist therapy duration
	At distraction surgery (cm)	15 days postoperatively (cm)				
Case 1	15 × 2	9.5 × 2	3	Distraction speed adjusted 0.5–1 mm/day starting 3 days post-surgery, completed in 3 sessions over 12 days; maintained for 5 days, total distraction duration 20 days	Silver ion dressing (15 days before distraction, 15 days after distraction); rh-bFGF and mussel adhesive dressing (started 15 days after distraction, used for 15 days); MEBO ulcer dressing (started 15 days after distraction, used for 20 days until wound closure)	40d
Case 2	Three sites, largest 3 × 1.8	2 × 0.8	1	Distraction speed adjusted 0.5–1 mm/day starting 3 days post-surgery, completed in 3 sessions over 9 days; maintained for 3 days, total distraction duration 15 days	Gentamicin gauze (14 days before distraction); Silver ion dressing (15 days before distraction, 20 days after distraction); rh-bFGF and mussel adhesive dressing (started 20 days after distraction, used for 15 days); MEBO ulcer dressing (started 25 days after distraction, used for 20 days until wound closure)	45d

## 3 Discussion

Identifying the underlying cause is a prerequisite and a key factor in developing a treatment plan (8). The treatment of exposed skull with chronic wounds faces multiple challenges, such as insufficient blood supply, high risk of infection, and poor tissue condition, which complicate and hinder the healing process. Therefore, successful repair not only relies on local wound management but also requires comprehensive and dynamic assessment of the patient’s overall condition and the wound’s microenvironment. This includes evaluating local blood supply, infection control, nutritional status, and the management of comorbidities. Given these challenges, it is essential to develop an individualized treatment plan and adjust it in a timely manner based on the changes observed during the wound healing process, thereby improving treatment success and healing rates. In this study, both patients received targeted cancer therapies. Case 1 was treated with everolimus and axitinib in phases, while Case 2 was treated with osimertinib and bevacizumab in phases. Several studies have shown that targeted therapy drugs may delay wound healing by affecting mechanisms such as cell proliferation, angiogenesis, and inflammatory responses (9–11). Case 2, who underwent head radiation therapy and had an MRSA infection, experienced decreased granulation tissue formation and insufficient blood supply. Therefore, treatment included the application of gentamicin, metronidazole, and dexamethasone for topical wound care, with targeted therapy temporarily discontinued.

The inherent elasticity and biological extensibility of the skin provide the histological basis for its ability to be subjected to tension. The scalp is thick and dense, with the superficial layer firmly connected to the subcutaneous tissue and galea aponeurotica, forming a tri-layer structure that is difficult to separate. Once ruptured, the tissue tends to retract, and the formation of sinus tracts can extend deep to the periosteum, making traction more challenging (12). The elasticity and extensibility of scalp skin are relatively poor, and it is prone to damage when excessive tension is applied, increasing the difficulty of traction therapy. A review highlighted that the complication rate of tissue expanders used on the scalp can be as high as 27.3% (13), with some complications being catastrophic (14). In current clinical practice, the standard distraction rate during bone transport or limb deformity correction is approximately 1 mm/day. However, in this study, both patients presented with fragile wound margins and pre-existing scar tissue. Excessive or overly rapid distraction may lead to local tissue hypoxia and even skin necrosis. Therefore, we adopted a phased strategy to regulate the wound microenvironment—combining infection control, tension reduction, and regenerative promotion—to achieve wound closure through synergistic effects: (1) Infection Control Phase: Debridement was performed, and silver ion dressing (Bohmann Wet Silver, Germany) was applied to prevent infection through its antibacterial effect and to fill any sinus tracts. (2) Mechanical Distraction Phase: The Sustained Skin Distraction device (SSD; Henan Keke Biotechnology Co., Ltd., China) was applied on day 3 post-installation. The nut was rotated clockwise to gradually reduce mechanical stress at the wound edges, with distraction adjusted at a rate of 0.5–1 mm/day over three sessions. If the patient experienced tightness or pain during distraction, the rate was slowed or paused, and wound edge perfusion was monitored. In this study, both patients had undergone multiple prior surgeries, resulting in scar formation and fragile wound edges prone to rolling and retraction, which could not tolerate rapid or excessive traction or subsequent suturing. In particular, for patient 2, granulation tissue grew slowly, and the wound edges retracted quickly after SSD placement. (3) Closure and Repair Phase: Recombinant human basic fibroblast growth factor (rh-bFGF; Gaifu, Nanhai Longtime Pharmaceutical Co., Ltd., China) was used to counteract the inhibitory effects of targeted therapies (e.g., anti-angiogenic agents) on fibroblasts and stimulate cell regeneration. Mussel adhesive wound repair dressing (Youbeiruikang, Jiangyin Beirisen Biochemical Technology Co., Ltd., China) provided antibacterial protection, inhibited biofilm formation, and maintained a moist environment, creating a synergistic effect of “promoting repair + controlling infection.” Once infection was controlled, necrotic tissue removed, and the wound appeared healthy, silver ion dressings were discontinued, and rh-bFGF and mussel adhesive dressings were applied. MEBO ulcer dressing, containing ingredients such as sesame oil and beeswax, was overlaid to maintain a moist environment and protect against external contamination. An outer layer of self-adhesive elastic bandage was used to approximate the wound edges and maintain reduced tension, ultimately achieving wound closure. Consolidation Phase: One week after complete wound closure, silicone dressings were applied for scar prevention for 3 months, improving scar elasticity, reducing traction on surrounding tissue, restoring partial skin function, and continuing to monitor the effects of targeted therapy on the skin. The combined use of multiple moist dressings played a critical role in maintaining a moist environment, promoting tissue regeneration, and controlling infection. Through precise selection of dressings during distraction therapy, the conditions for wound edge growth were optimized, providing an environment conducive to healing.

In addition, the treatment of complex head wounds relies not only on technical measures but also on multidisciplinary collaboration involving medical, nursing, wound care, and nutrition teams, forming a dynamic and closed-loop management system (7). In this case, the primary role of the physician focused on the initial diagnosis of the wound, formulation of the treatment plan, and surgical operation, while the wound care nurse, in addition to performing routine daily care, was responsible for the continuous care and dressing changes of the wound. Clinical management also required a focus on infection control, pharmacological interventions, and dynamic assessment of the wound edge’s blood supply (15, 16). Meanwhile, the active participation of the patient played a crucial role in the treatment process. By understanding and engaging in the treatment plan, patients and their families not only increased their involvement in medical decision-making but also enhanced their confidence in and compliance with the treatment. Combining comprehensive, multidisciplinary interventions and collaboration improves the effectiveness of treating complex wounds, significantly shortening the healing time, reducing complications, and ultimately improving patient prognosis. In this study, the patients were followed up for 6 months, and neither of the two patients experienced any adverse events or related complications.

## 4 Limitations

This study includes only two cases from a single center, which limits the generalizability of the findings. The small sample size and single-center design may introduce bias, and further studies with larger cohorts are warranted to validate the observed outcomes.

## 5 Conclusion

This study demonstrates that the staged application of sustainable SSD combined with moist dressings effectively integrates mechanical tension and biological repair, potentially offering an effective minimally invasive treatment option for the repair of skull exposure with chronic non-healing wounds. By dynamically adjusting the tension and improving the wound microenvironment, this approach helps promote the extension of wound edges and spontaneous wound closure, warranting further investigation to validate its efficacy and safety.

## Data Availability

The original contributions presented in the study are included in the article/supplementary material, further inquiries can be directed to the corresponding authors.
